# Genotype-phenotype correlation of deletions and duplications of 4p: case reports and literature review

**DOI:** 10.3389/fgene.2023.1174314

**Published:** 2023-06-14

**Authors:** Xuan Zhang, Hongjuan Lu, Hanran Yang, Yichen Ji, Huixin Liu, Wenjian Liu, Jiayi Li, Zhixian Yang, Wei Sun

**Affiliations:** ^1^ Department of Neurology, Xuanwu Hospital, Capital Medical University, Beijing, China; ^2^ Department of Pediatrics, Peking University First Hospital, Beijing, China

**Keywords:** chromosome 4p, terminal 4p deletion, wolf-Hirschhorn syndrome, partial duplication 4p, 4p trisomy

## Abstract

Structural rearrangements of chromosome 4p gives rise to a group of rare genomic disorders that mainly result in two different clinical entities: Wolf-Hirschhorn syndrome (WHS) and partial 4p trisomy. The severity of the phenotype depends on the size of the deletion or locus duplication. Here, we present two unrelated individuals with a copy number variation of chromosome 4p. Inverted duplication deletions (inv dup-del) in 4p are particularly rare. Case 1 describes a 15-year-old girl with a 1.055 Mb deletion of terminal 4p, distal to the recognized critical region of WHS, and a large duplication of 9.6 Mb in size from 4p16.3 to p16.1. She had postnatal development delay, intellectual disability (especially pronounced in speech), seizure/electroencephalogram anomalies, and facial dysmorphic features. This unusual chromosomal imbalance resulted in the WHS phenotype rather than the 4p trisomy syndrome phenotype. Case 2 describes a 21-month-old boy with a 1.386 Mb terminal 4p deletion who presented with slight developmental delay, borderline intellectual disability, and seizures. Combined with previous reported cases of 4 pter del-dup or pure 4p terminal deletions, our observations suggest that terminal chromosome 4p deletion is more pathogenic than the concomitant partial 4p duplication, and some regions of the 4p terminal may have regulatory effects on the remaining region of 4p. About nine cases have been reported thus far to date, and our study delineates further genotype-phenotype correlations about terminal 4p duplication-deletions for predicting disease prognosis and patient counseling.

## 1 Introduction

The short arm of chromosome 4 (4p) is approximately 50.86 Mb in size and contains approximately 379 genes, approximately 45% of which are located 10 Mb distal to 4p (Hannes et al., 2008). Rearrangements of chromosome 4p mainly cause two different clinical entities: partial 4p trisomy and Wolf-Hirschhorn syndrome (WHS). Wilson et al. (1970) first described 4p trisomy, which is associated with large duplications containing 2/3 of chromosome 4p. 4p trisomy is characterized by a constellation of facial dysmorphic features, prenatal and postnatal growth retardation, intellectual disability, cardiac anomalies, and renal anomalies. The dysmorphic features include a prominent glabella, bulbous nose with a flat nasal bridge, retrognathia and malformed ears with abnormal helix and antihelix.

In the 1960s, Wolf et al. (1965) and Hirschhorn et al. (1965) published the first patients with WHS. WHS is a multiple congenital anomaly syndrome, with clinical manifestations including pre- and postnatal growth retardation, intellectual disability, seizures (or EEG anomalies), major malformations and a characteristic facial anomaly of a broad nasal bridge, microcephaly, high forehead with prominent glabella, ocular hypertelorism, epicanthus, highly arched eyebrows, short philtrum, downturned corners of the mouth, micrognathia, and hypoplastic ears with pits or tags. Prominent forehead, hypertelorism, and the wide bridge of the nose continuing to the forehead lead to the appearance of a typical ‘Greek warrior helmet’. Major malformations include congenital heart defects, ocular colobomas, renal abnormalities, skeletal abnormalities and midline defects such as cleft palate and hypospadias (Nevado et al., 2020). WHS is also classified as a contiguous gene syndrome and is usually caused by partial deletion of terminal 4p. Wieczorek et al. (2008) and Zollino et al. (2008) found that the severity of WHS clinical characteristics, such as craniofacial anomalies and intellectual disability, were positively correlated with the length of the 4p terminal deletion. Thus, different lengths of 4p deletions may contribute to the heterogeneity in clinical presentations of WHS patients.

**FIGURE 1:**
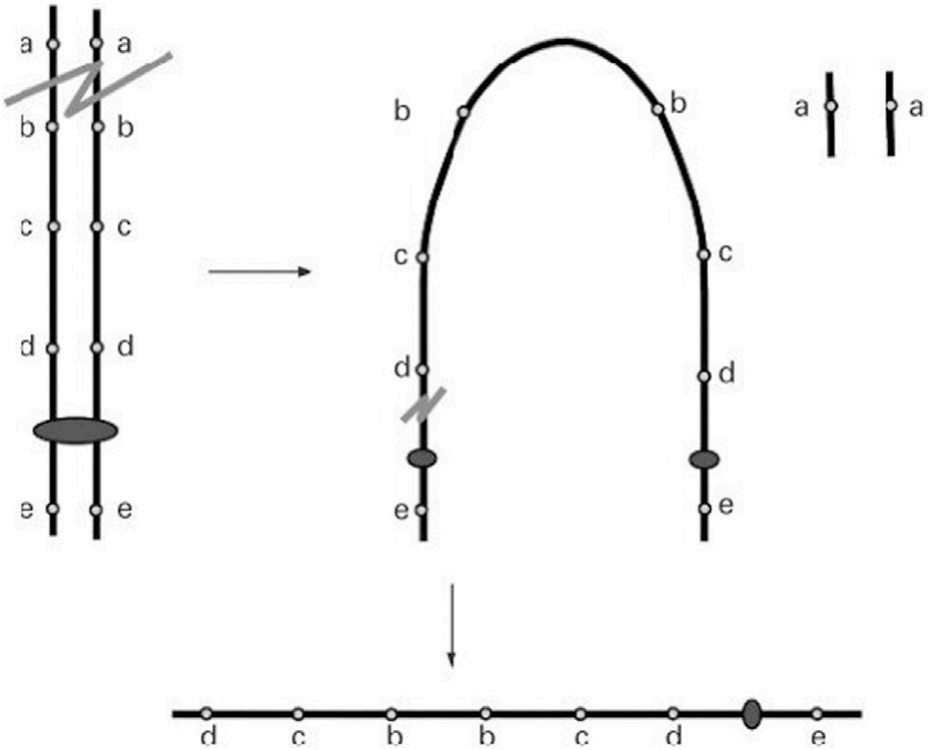
A double-strand break of one chromosome is repaired by fusion of the two sister chromatids (U-type exchange) which results in a dicentric chromosome. Pre-zygotic breakage of the dicentric outside the fusion region leads to a monocentric chromosome with a terminal deletion of the ‘‘a’’ locus and an inverted duplication of the ‘‘b’’, ‘‘c’’, and ‘‘d’’ loci with no single copy region between the duplication (L R Rowe et al., 2014).

Two critical regions have been identified as candidate regions underlying WHS pathogenesis. The first is WHSCR, which is limited to a 165 kb interval around 1.9–2.1 Mb from the telomere. The second is WHSCR-2, which lies within a 300–600 kb interval 1.6–1.3 Mb from the telomere and is contiguous distally to WHSCR. Most patients carry a terminal deletion, and haploinsufficiency of these genes located in the critical region contributes to the phenotypic features of WHS. Simple 4p deletions, whether they are terminal or interstitial deletions, account for about 70% of all WHS cases. Approximately 22% of cases have an unbalanced translocation, and the remaining cases have complex rearrangements, such as inverted duplications (Battaglia et al., 2015). Cases of 4p inverted duplication deletions (inv dup-del) are rare and heterogeneous. Thus far, only nine patients have been reported. The proposed mechanism of Inv del-dup includes terminal deletion with two chromatid breaks, followed by re-connection of the broken ends on the chromatids after DNA replication, like a U type, to form a dicentric intermediate (Figure 1) (Gordon and Halliday, 1995; L R Rowe et al., 2014).

Here, we report two cases of rearrangements of chromosome 4p. Cases with inverted duplication of distal 4p accompanied by a subtelomeric deletion are rare, and our case provides further information relevant to the clinical phenotype and genotype of the deletion and duplication of 4p.

## 2 Case description

### 2.1 Case 1

**FIGURE 2:**
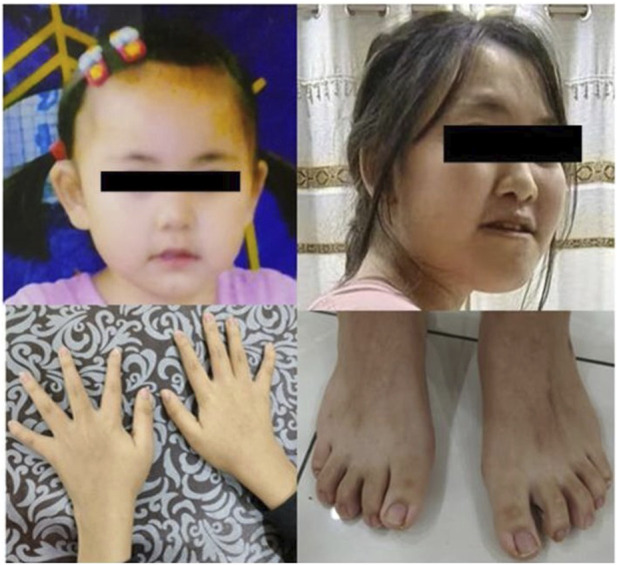
The picture of the patient (case 1). The upper picture is when the patient is at 2 years old and 15 years old, respectively. Below are photos of the patient’s hands and feet.

The patient was a 15-year-old girl who was the second child of apparently healthy, non-consanguineous parents, with no similar cases in the family. Her mother was pregnant and gave birth at 36 years of age. The pregnancy was uneventful, and there was no teratogenic exposure. Height, weight, and head circumference measurements were all around the 50th percentile since birth. She had no hypotonia. The facial characteristics (Figure 2) were unremarkable, and were limited to large and protruding eyes, high nasal bridge, short philtrum, downturned corners of the mouth, and low-set and large ears. The patient’s developmental milestones were delayed. Gross motor development was slightly delayed, and language was also delayed and limited to single words or short sentences. She began to have febrile seizures at the age of 8 years, myoclonic seizures which lasted for 9 years, and was seizure-free after valproic acid administration. EEG examination showed normal background activity and spike-wave or poly-spike-wave discharges in the central and posterior cortical regions during waking and sleep. She had learning difficulties and her intellectual performance appeared to be within the lower limits. Although she had attended a normal school, her academic difficulties resulted in her academic performance always being lower than that of her colleagues. She was diagnosed with scoliosis, bilateral clinodactylous of the index and fifth fingers, and flat feet with bilateral clinodactyly of toes 2 and 5. The teeth in this patient were sparse. The patient had normal hearing and vision. Magnetic resonance imaging revealed a left temporal arachnoid cyst with abnormal subcortical signals in the left frontal lobe and right parietal lobe. The patients’ cardiac and renal examinations were normal.

### 2.2 Case 2

A 21-month-old boy was born at full term via cesarean section after an uneventful pregnancy. He is the second child of an apparently healthy, non-consanguineous parent, with no similar cases in the family. His birth weight, length, and head circumference were not recorded. Motor and language development were slightly delayed. The patient’s facial characteristics were unremarkable. He had complex febrile seizures at 10 months of age and daily seizures characterized by eyelid myoclonus and drop attacks lasting for 2–3 s, occurring more than 10 times a day. EEG examination showed generalized spike-wave discharges. Cardiac and renal examinations were normal.

### 2.3 Clinical examinations of the patients

Peripheral blood samples were obtained from the two patients and their parents. DNA was extracted from the patients peripheral blood samples, then, DNA was fragmented, and DNA libraries were constructed by end filling, adapter ligation and polymerase chain reaction (PCR) amplification. DNA libraries were subjected to massively parallel sequencing on the NovaSeq6000 (Illumina, San Diego, CA, United States) to generate raw sequencing reads. The TruSeg Library Construction Kit was used to build the database and the paired-end sequencing strategy was performed according to the manufacturer’s instructions. For each sample, raw reads were filtered to remove low quality reads by using FastQC, high-quality sequences were obtained (Q30 > 80%) from the raw data. Clean reads were mapped to the reference genome GRCh37/hg19 to obtain bioinformatic results and determine the existence of chromosomal variation and CNVs. The Database of Genomic Variants, DECIPHER database, ClinVar, OMIM, and ClinGen were used for interpretation and classification of the clinical significance of candidate CNVs according to previously reported guidelines.

### 2.4 Results

**FIGURE 3:**
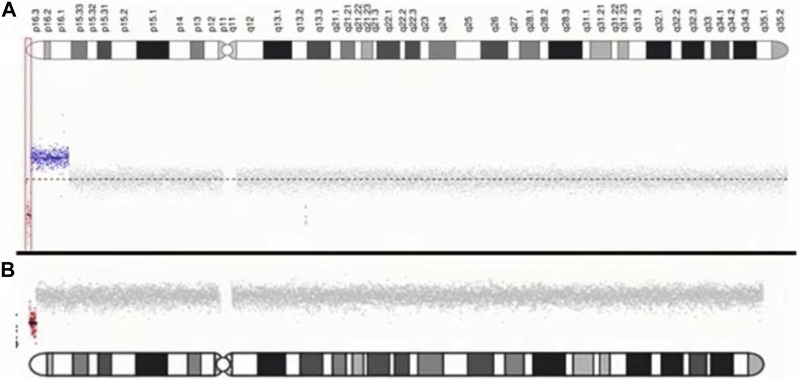
Characterization of chromosome 4p of two patients. **(A)** case1: The red dot indicates the location of the submicroscopic deletion. The blue dot indicates the location of the submicroscopic duplication. **(B)** case2: The red dot indicates the location of the submicroscopic deletion.

For case 1, CNV-seq results showed a 1.055 Mb deletion of terminal 4p and a contiguous large duplication of 9.6 Mb in size extending from 4p16.3 to p16.1. The patient in case 2 was found to carry a 1.386 Mb terminal deletion of 4p using CNV-seq. The results of genomic maps can be seen in Figure 3.

## 3 Discussion

We have reported two cases with a 1.055 Mb 4p terminal deletion, a contiguous large duplication of 9.6 Mb, and a pure 1.386 Mb 4p terminal deletion. Case 1 was a girl with a mild WHS phenotype, including mild facial anomalies, epilepsy, skeletal anomalies, mild postnatal developmental delay, and intellectual disability, especially in speech, while clinical manifestations of 4p trisomy syndrome were practically absent. This patient carried an inverted duplication for 4p16.3-p16.1, a deletion of 4p16.3-4-pter, a 1.055 Mb 4p terminal deletion, distal to the critical region of WHS, and a large duplication of 9.6 Mb in size from 4p16.3 to p16.1, comprising the WHS critical region.

To date, patients with inverted terminal deletion duplication of 4p are relatively rare, and approximately nine cases have been reported (Table 1). Among them, two 4 pter del-dup cases were detected prenatally, and the pregnancies were terminated at 14 and 36 weeks, respectively. To demonstrate the roles of partial 4p deletion and 4p duplication in the phenotypic features of patients, we compared the clinical presentation of patients carrying a 4 pter del-dup with patients carrying similar pure 4p terminal deletions (Table 2). The phenotype of patients with a 4 pter del-dup was generally consistent with that of patients carrying a similarly sized pure 4p deletion. In groups 1, 2, 3, and 6, patients carried a relatively large deletion of terminal 4p, encompassing the accepted critical region of WHS, which appeared to be more consistent with WHS syndrome. Seizure onset in WHS generally occurs within the first 2 years of the patient’s life with a peak incidence at 6–12 months of age; therefore, patients with pure 4p deletions in groups 3 and 6 were too young to develop seizures (Battaglia & Carey, 2005). The size of the deletion seems to be more prognostic of phenotypic severity than the size of the duplication.

**TABLE 1:** Summary of the clinical features of the cases with inv del-dup of 4p in literatures.

Clinical sign	Present case1	Zollino 1999	Cotter 2001	Kondoh 2003	Beaujard2005	Berg 2007	Paskulin 2008	Roselló 2009	Piccione 2015	Fontana 2020
Age/sex	15Y/F	5Y/M	4Y/F	8Y/F	36W^a^/F	5Y/M	4Y/F	9Y/M	3Y/M	14W^b^/M
Deletion location (size (Mb))	4p16.3pter (1.055)	4p16.3pter (3.2)	4p16.3pter (0.7)	4p16.3pter (2.8)	4p16.1pter (10)	4pterp16.1 (8.3)	4p16.3pter (0.52)	4p16.3pter (1.3)	4pterp16.1 (8.6)	4p16.3pter (0.6)
Duplication location (size (Mb))	4p16.1p16.3 (9.6)	4p16.1p16.3 (1.7)	4p16.3p15.3 (9.3)	4p16.3p14 (33.2)	4p14p16.1 (30)	4p15.33p16.1 (2.6)	4p16.3p13 (41.65)	4p16.3 (1.1)	4p16.1p15.32 (6.7)	4p16.3p15.2 (21.6)
intellectual disability	+	+	+	+		+	+	+	+	
Hypotonia	+	+	+	+		+	+	+	+	
growth retardation	+	+	+	+	+	+	+	+	+	+
Prenatal growth		+			+	+	+		+	+
Postnatal growth	+	+	+	+		+	+	+	+	
Epilepsy/EEG anomalies	+	+	+	+		+	+	+	+	
WHS facial appearance	+	+	−	+	+	+	+	+/−	+	
Ears anomalies	+	+	+	+	+	+	+	+	+	
Cleft lip and/palate	−	−	−	−	+	−		−		
Microcephaly	+/−	+	+	+	+	+	+	−	+	
Ophthalmologic	−	−	NA	NA		+	+	NA	−	
Renal anomalies	−	−	NA	NA	+	+	+	−	−	+
CHD	NA	−	NA	NA		−	+	−	+	+
Skeletal anomalies	+	+	+	+	+	NA	+	−	−	+
Dermatoglyphics	NA	+	NA	NA		NA	NA	NA	NA	
HG	NA	+	NA	NA	+	+		NA	NA	+
Other complication(s)			DVS	MDM	HCC, PM, FCD, LH, TH	HCC, DM	DVSD, HGA	BD, UA	HGA, HCC, MDM, ELS in FL	LH, GS, IA

Y, year-old; M, male; F, female; CHD, congential heart defects; HG, Hypoplastic genitalia/hypospadias; DVS, dilatation of the ventricular system; MDM, mild delay of myelination; HCC, hypoplasia of the corpus callosum; PM, polymicrogyria; FCD, focal cortical dysplasia; DM, diabetes mellitus; HGA, hemangioma; BD, behavioral disorders including hyperactivity, impulsivity, and aggressive bursts; UA, unspecific signal alteration in the brain; ELS in FL, a moderate enlargement of the liquoral spaces of the frontal lobe; LH, lung hypoplasia; GS, gastroschisis; IA, imperforate anus; TH, thymic hypoplasia.

^a^ Pregnancy was terminated at 36 weeks.

^b^ Pregnancy was terminated at 14 weeks.

**TABLE 2:** Comparison of phenotypes in the cases carrying inv dup-del or pure del of 4p.

	1		2		3		4	
	Zollino et al. (1999)	Faravelli et al. (2007)	Kondoh et al. (2003)	Maas et al. (2008)	Flipsen-ten Berg et al. (2007)	Yang et al. (2016)	Roselló et al. (2009)	Case 2
Deletion location (size (Mb))	4p16.3pter (3.2)	4p16.3pter (3.2)	4p16.3pter (2.8)	4p16.3pter (2.7)	4pterp16.1 (8.3)	4pterp16.1 (8.35)	4p16.3pter (1.3)	4p16.3pter (1.3)
Duplication location (size (Mb))	4p16.1p16.3 (1.7)		4p16.3p14 (33.2)		4p15.33p16.1 (2.6)		4p16.3 (1.1)	
Age/Gender	5Y/M	18Y/M	8Y/F	3Y/F	5Y/M	4M/M	10Y/M	21M/F
Growth delay	IUGR, PGD	IUGR, PGD	PGD	IUGR, PGD	IUGR, PGD	PGD	PGD	PGD
Microcephaly	+	+	+	+	+	NA	−	−
Facial appearance	large and protruding eyes, high nasal bridge, short philtrum, prominent upper lip, large anteverted and low-set ears, downturned corners of mouth	hypertelorism, large and protruding eyes, high nasal bridge, short philtrum, prominent upper lip, downturned corners of mouth, cleft palate	prominent grabella, large and malformed ears, a nose with bulbous tip, hypoplastic alae nasi, high and wide nasal root, a short philtrum, a small mouth with downturned corners, high-arched palate, micrognathia	high nasal bridge, high forehead, hypertelorism, Prominent glabella, broad nose, epicanthus, short philtrum, downturned shape of the mouth, dysplastic ears	broad nasal bridge, hypertelorism, epicanthal folds, coloboma of the eye	broad forehead, prominent glabella, hypertelorism, prominent or slanting eyes, high arched eyebrows, broad nasal bridge, short philtrum	hypertelorism, epicanthal folds, broad nasal bridge, low-set ears, downturned corners of the mouth	Hypertelorism, broad nasal bridge, low-set large ear
Intellectual disability	+	+	+	+	+	+	+	+ Borderline
Epilepsy/EEG anomalies	+	+	+	+	+	−	+	+
Renal anomalies	−	−	NA	−	+	−	−	−
CHD	−	−	NA	−	−	+	−	−
Skeletal anomalies	+	−	+	−	NA	−	−	−
Dermatoglyphics	+	NA	NA	NA	NA	NA	NA	NA
HG	+	+	NA	−	+	NA	NA	NA
Other complication(s)			MDM		HCC, DM		BD, UA	

	5		6		7		
	Paskulin et al. (2009)	Zollino et al. (2014)	Piccione et al. (2015)	Sheth, Akinde, Datar, Adeteye, & Sheth (2012)	Cotter et al. (2001)	Case 1		Faravelli et al. (2007)
Deletion location (size (Mb))	4p16.3pter (0.52)	4p16.3pter (0.56)	4pterp16.1 (8.6)	4pterp16.1 (8.5)	4p16.3pter (0.7)	4p16.3pter (1.05)		4p16.3pter (1.5)
Duplication location (size (Mb))	4p16.3p13 (41.65)		4p16.1p15.32 (6.7)		4p16.3p15.3 (9.3)	4p16.3p16.1 (9.6)		
Age/Gender	4Y/F	6Y/F	3Y/M	8M/M	4Y/F	15Y/F		56Y/F
Growth delay	IUGR, PGD	PGD (slight)	IUGR, PGD	IUGR, PGD	PGD	PGD		−
Microcephaly	+	−	+	+	+	−		HC:55 cm
Facial appearance	high forehead, frontal bossing, epicanthal folds, strabismus, nystagmus, low nasal bridge, wide nasal bridge, bulbous nasal tip, long philtrum, downturned corners of the mouth	protruding eyes, down-slanting palpebral fissures, arched eyebrows, and high nasal bridge	broad forehead, hypertelorism, hypoplastic nose with depressed root, small mouth, downturned corners of the mouth, micrognathia	prominent glabella, short philtrum, micrognathia, high forehead, preauricular tags with low set ears, narrow external auditory canals, strabismus, hypertelorism, iris coloboma, wide nasal bridge, downturned corners of the mouth	prominent glabella, deep-set eyes, bulbous nose, abnormal lobulation of the ears, a high arched palate	large and protruding eyes, high nasal bridge, short philtrum, downturned corners of mouth, low-set and large ears		hypertelorism, high nasal bridge, protruding eyes, down slanting palpebral fissures
Intellectual disability	+	+(Borderline)	+	+	+	+(Borderline)		+(Borderline)
Epilepsy/EEG anomalies	+	+	+	−	+	+		+
Renal anomalies	+	NA	−	−	NA	−		NA
CHD	+	NA	+	+	NA	NA		NA
Skeletal anomalies	+	NA	−	−	+	+		NA
Dermatoglyphics	NA	NA	NA	NA	NA	NA		NA
HG	NA	NA	NA	−	NA	NA		NA
Other complication(s)			HGA, HCC, MDM, ELS in FL		DVS			

Y, year-old; M, male; F, female; IUGR, intrauterine growth restriction, PGD, postnatal growth delay; CHD, congenital heart defects; HG, Hypoplastic genitalia/hypospadias; DVS, dilatation of the ventricular system; MDM, mild delay of myelination; HCC, hypoplasia of the corpus callosum; PM, polymicrogyria; FCD, focal cortical dysplasia; DM, diabetes mellitus; HGA, hemangioma; BD, behavioral disorders including hyperactivity, impulsivity, and aggressive bursts; UA, unspecific signal alteration in the brain; ELS in FL, a moderate enlargement of the liquoral spaces of the frontal lobe; LH, lung hypoplasia; GS, gastroschisis; IA, imperforate anus; TH, thymic hypoplasia; HC, head circumference.

**FIGURE 4:**
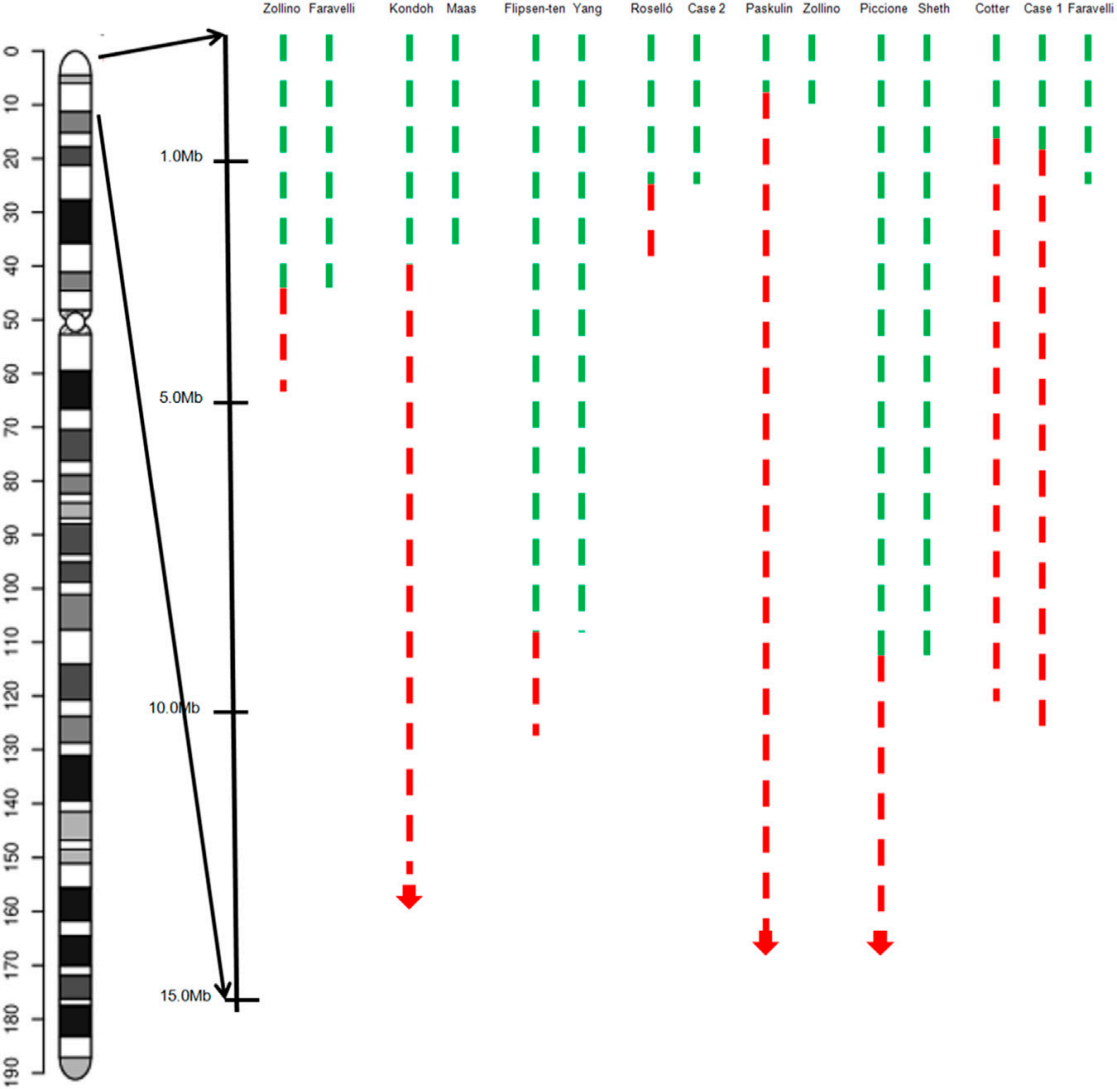
Graphical representation of the overview of the molecular cytogenetic region involved in a case with inv del-dup of 4p and pure terminal deletion of 4p in the literature. Green dashed lines indicate the deleted segments on chromosome 4p, red dashed lines the duplicated regions.

Two seizure-susceptibility regions have been identified in WHS. The distal candidate region is a 197 kb segment starting from 368 kb to 565 kb of the terminus of 4p, while the proximal seizure candidate region maps from 0.76 to 1.3 Mb from the end of 4p terminus (South et al., 2008). Almost all the patients suffered from seizures and had a deletion encompassing both distal regions. There is an overlap region of 0.5 Mb from terminal between these patients. It has been reported that 4p terminal deletions between 200 and 400 kb are likely benign (Hannes & Vermeesch, 2008). Therefore, we propose that the region spanning 0.4–0.52 Mb from the terminus of 4p is a seizure candidate region (Figure 4) and a total of 3 RefSeq genes reside within this region. Only PIGG (phosphatidylinositol glycan anchor biosynthesis, class G; PIGG) are in the OMIM database and have already been proposed as candidate genes for seizures. PIGG is a glycosylphosphatidylinositol that anchors proteins to the cell surface. Homozygous and compound heterozygous variants of PIGG have been reported in five patients with intellectual disability, hypotonia, and early onset seizures, whereas parents and siblings with heterozygous variants of PIGG were asymptomatic carriers (Makrythanasis et al., 2016; Corrêa et al., 2022). This means that haploinsufficiency for PIGG is not sufficient to develop seizures, and several genes may function synergistically to promote seizures. In addition, we believe that this region may be associated with mild or borderline intellectual disability, notably speech delay.

Several patients with 4 pter del-dup reported by Paskulin et al. (2009) and Cotter et al. (2001) carried a deletion of terminal 4p, distal to the critical region, and presented with characteristics corresponding to 4p trisomy syndrome. Cotter et al. (2001) reported a 4-year-old girl with a 4p terminal deletion about 0.7 Mb distal to the WHS critical region and a 9.3 Mb duplication from 4p16.3 to p15.3, giving rise to typical findings of 4p trisomy including prominent glabella, bulbous nose, multiple congenital anomalies, seizure disorder, and severe intellectual disability. A 3.2 Mb 4p terminal deletion and 1.7 Mb duplication was detected in another patient diagnosed with WHS. Zollino et al. (1999) speculated that the critical WHS region plays a role as a ‘‘master’’ regulatory sequence, with strong pleiotropic effects on the other genes in a dose-dependent manner. Our patient and the patient reported by Roselló et al. (2009) carried a 1.055 Mb and 1.3 Mb 4p terminal deletion, respectively, and presented with a more pronounced WHS syndrome phenotype. Hannes et al. (2010) reported a 7-year-old boy clinically diagnosed with mild WHS features, and molecular karyotyping revealed a deletion 600 kb proximal to WHSCR and WHSCR-2. They suggested that the WHSCR1-2 flanking sequence directly or indirectly contributes to the severity of WHS. South et al. (2008) also found that WHS patients with 4p deletions flanking the WHSCR display a specific facial gestalt. So, combined with our patients, we speculate that the region 0.7 Mb–1.3 Mb from the 4p terminus may harbor controlling genes that regulate the expression of genes in the rest of the 4p region, especially in the accepted WHS critical region (Figure 3).

Although partial 4p trisomy and WHS are two different clinical entities, there is overlap in the clinical phenotype between pure duplication starting from the 4p terminal (as in 4p trisomy), and pure deletion of the 4p terminal (as in WHS). Both are characterized by similar features, such as developmental and neuropsychomotor delays, especially predominant in speech delay, as well as microcephaly, low weight, wide nasal bridge/root, dysplastic ears, and other organ anomalies. Two interstitial microduplications encompassing the WHS critical region have been reported (between 1.3Mb and 1.9 Mb from terminal 4p), both have overgrowth features such as macrocephaly, overgrowth in height, and bone age (Cyr et al., 2011; Palumbo et al., 2015). None of these phenotypes were found in the nine patients mentioned above or in the two cases discussed in the present study. This study also demonstrated that particular genes or regions located in the 4p terminal region, like 0.7 Mb–1.3 Mb from the 4p terminus, have a regulatory influence on the critical region of WHS.

Genotype-phenotype correlations contribute to the identification of genes involved in the pathogenesis of genetic syndromes. Combined with previously reported cases, we found that the region 0.7 Mb–1.3 Mb from the 4p terminus may have a regulatory effect on the rest of the 4p region, especially in the accepted WHS critical region, and has a critical effect on patient phenotypes. Structural rearrangements of chromosome 4p are complicated, and in cases of 4p inv dup-del, the phenotypes are heterogeneous. More accurate clinical descriptions of patients need to be reported, and the roles and functions of genes located on 4p need to be elucidated.

## Data Availability

The original contributions presented in the study are included in the article/Supplementary material, further inquiries can be directed to the corresponding author.
